# A Rare Disease Presenting Postpartum: Acute Fatty Liver of Pregnancy

**DOI:** 10.1155/2021/1143470

**Published:** 2021-09-30

**Authors:** Sofía Córdoba-Vives, Patricia Pérez-Rodríguez, Astrid Marín-Delgado, Miguel Matus-Vargas, Adriana Arias-González

**Affiliations:** ^1^Department of Maternal Fetal Medicine, Rafael Angel Calderón Guardia Hospital, San José, Costa Rica; ^2^University of Costa Rica, San José, Costa Rica; ^3^Department of Obstetrics and Gynecology, Rafael Angel Calderón Guardia Hospital, San José, Costa Rica; ^4^Department of Pathology, Rafael Angel Calderón Guardia Hospital, San José, Costa Rica

## Abstract

Acute fatty liver of pregnancy is a rare but highly fatal disease affecting women most frequently during the third trimester of pregnancy or in the postpartum period. It is considered a diagnosis of exclusion and requires a timely diagnosis to avoid maternal mortality. We present the case of a 33-year-old primigravida who required an emergency cesarean section due to fetal bradycardia. On postoperative day one, the patient was noted jaundiced, oliguric, and hypoglycemic. Laboratory tests revealed important hepatic dysfunction, coagulopathy, and renal failure. She was admitted to the Intensive Care Unit with the suspicion of acute fatty liver of pregnancy. Plasma exchange was started on postoperative day 5 with major clinical and laboratory improvement. A transjugular hepatic biopsy confirmed the diagnosis. The patient had satisfactory evolution and was discharged 15 days after delivery. Acute fatty liver of pregnancy is a highly morbid disease that needs a high index of suspicion to be diagnosed. Admission to an Intensive Care Unit to ensure maximum supportive care is mandatory in this disease.

## 1. Introduction

Acute fatty liver of pregnancy (AFLP) is a rare but highly morbid disease. The majority of AFLP cases are diagnosed in the third trimester of pregnancy or during the early postpartum period with some isolated reports occurring in the second trimester of pregnancy [1]. The disease is characterized by jaundice, coagulopathy, and signs and symptoms of hepatic and renal failure [2]. Diagnostic criteria for AFLP, known as the Swansea criteria, have been proposed to distinguish AFLP from other causes of liver dysfunction, including HELLP syndrome [3]. Accurate and timely diagnosis is fundamental to ensure good maternal and fetal outcomes.

In the last years, maternal mortality has been reduced due to the implementation of new therapies such as artificial liver support therapy (ALST). We present the case of a patient presenting with AFLP in the immediate postpartum who was admitted to the Intensive Care Unit (ICU) and received plasma exchange (PE) with excellent results.

## 2. Case Report

A 33-year-old primigravida consulted the emergency department with a 40-week pregnancy and history of spontaneous rupture of membranes (SROM). The diagnosis of SROM was confirmed, and she was admitted to labor and delivery. Augmentation of labor was initiated, and approximately 2 hours after admission, fetal bradycardia was observed, and she was taken to an emergency cesarean delivery. During surgery, about 50% of placental abruption was diagnosed. No important bleeding was reported afterwards, and no surgical complications were reported. A healthy female newborn was obtained, who weighed 3.3 kgs, Apgar score was 8-9, and no neonatal complications were reported.

Fourteen hours after the surgery, the patient was noted jaundiced, oliguric, and hypoglycemic (glycemia: 40 mg/dL). She was conscious and alert, and no important obstetric bleeding was evidenced at the time. Her vital signs at that moment were the following: heart rate: 120 beats per minute (BPM), peripheral capillary oxygen saturation (SpO_2_): 95%, respiratory rate: 16 respirations per minute (RPM), temperature 36.3 degree Celsius, and blood pressure: 74/48 mmHg.

Laboratory tests evidenced important hepatic dysfunction (Total Bilirubin (TB) 7.9 mg/dL, Indirect Bilirubin (IB) 2.7 mg/dL, direct bilirubin (DB) 5.149 mg/dL, Aspartate Aminotransferase (ASAT) 170 U/L, and Alanine Aminotransferase (ALAT) 149 U/L), coagulopathy (prothrombin time (PT) 27 seconds, International Normalized Ratio (INR) 2.56, and fibrinogen 71 mg/dL), acute kidney injury (creatine 2.7 mg/dL) as well as hyperammonemia (126 *μ*mol/L), low platelets (95 000), and leukocytosis (33 030/microliter). At this point, her hemoglobin level was 9.9 mg/dL, hematocrit 27.8%, and lactate dehydrogenase (LDH) 432 IU/L. Due to the laboratory anomalies, an abdominal ultrasound was ordered. The ultrasound revealed an echogenic liver with fatty infiltrates and mild hepatic steatosis.

It is important to note that on the days prior to her hospital admission, the patient had not presented with any symptoms suggesting hepatic failure, and she had no known exposure to virus or medications that could explain the clinical alterations. At this moment, the patient was considered to have 7 Swansea criteria and was admitted to the ICU with suspicion of AFLP.

In the ICU, resolution of the coagulopathy was initiated with blood products. The transfusion was guided by rotational tromboelastography (ROTEM). She required platelets (1 pool), fresh frozen plasma (1 unit), and cryoprecipitates (1 unit). The patient remained stable for the next 3 days.

On postoperative day 5, the patient deteriorated importantly. She turned disoriented and encephalopathic; she was coagulopathic (prolonged PT and INR > 5) once more and persistently hypoglycemic despite an adequate diet. Other causes of hepatic failure in pregnancy and postpartum were discarded, such as viral hepatitis. At this point, the decision to start plasma exchange therapy was made, she received a total of three sessions (one daily) of PE, and recovery of her neurological status and laboratory tests was reached on postoperative day 9.

Once the coagulation disorder was reversed, she was taken to a transjugular hepatic biopsy. The biopsy reported microvesicular steatosis, ballooning degeneration of hepatocytes, canalicular cholestasis, and cholangiolitis, findings suggesting AFLP considering the clinical context. On postoperative day 15, the patient was discharged home, with complete resolution of symptoms and normalization of hepatic and renal functions.

## 3. Discussion

AFLP is a rare and serious liver pregnancy-related disease with an incidence of 1 per 6500 to 20 000 pregnancies [4]. Publications in the 1980s suggested mortality rates as high as 70%; more recent studies, suggest lower mortality rates: approximately 20% in some centers in low-income countries or even less than 10% in high-income countries. This reduction in mortality is due to the better understanding of the disease, recognition of milder presentations, early intervention and delivery, as well as aggressive management of complications, and the implementation of new treatment modalities, like artificial liver support therapy (ALST), which includes plasma exchange (PE) [5].

Perinatal mortality for infants born to affected mothers is highly variable. Data show a perinatal mortality rate of 10 to 20%, with the majority of reported cases caused by a stillbirth. Perinatal morbidity has been related to fetal acidosis and prematurity [5].

Pathogenesis of AFLP is not completely elucidated, but it is related to an abnormal beta-oxidation of fatty acids in fetal mitochondria that is probably caused by a genetic mutation in long-chain 3-hydroxyl coenzyme A dehydrogenase; this mutation contributes to microvesicular fatty infiltration of the liver of the mother [2].

Patients with AFLP typically present with a story of 1–2 weeks of nausea and vomiting; this prodromal symptom has an incidence that varies from 60 to 100% [6]. The clinical diagnosis is made by the application of the Swansea criteria (Table 1), which have a high negative predictive value. Six of the fourteen criteria are required to confirm the diagnosis of AFLP, once other causes of liver disease are excluded [3]. Although her clinical presentation was atypical, our patient had 7 Swansea criteria when the initial suspicion of AFLP was raised.

The definitive and confirmatory diagnosis of this clinical entity remains to be made histologically [7]. However, liver biopsy is not always performed routinely to establish the diagnosis of AFLP, due to coagulation disorders that usually manifest and the consequent bleeding complications that a patient undergoing the procedure could present [8]. To avoid these complications, in some hospitals, hepatic biopsies are done percutaneously, such is the case of our patient who underwent a transjugular hepatic biopsy.

**Figure 1:**
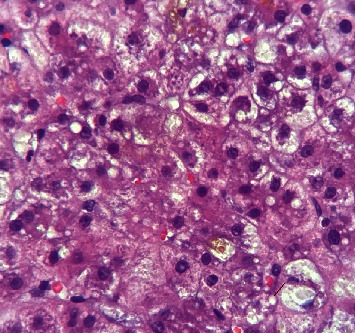
Microvesicular steatosis. Note the small white intracytoplasmic vesicles in the hepatocytes (400x, H&E staining). *Source: own*.

**Figure 2:**
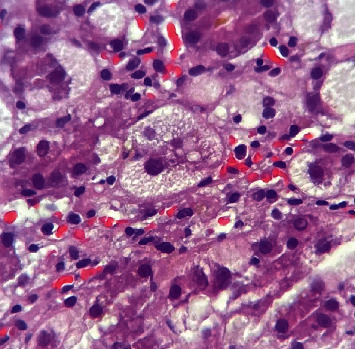
Ballooning degeneration. Note swollen hepatocytes with abundant and clear cytoplasm (400x, H&E staining). *Source: own*.

**Figure 3:**
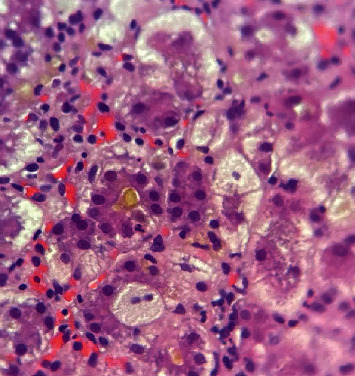
Canalicular cholestasis. Note the green-orange pigment located inside the canaliculus. On the right, ballooned hepatocytes (400x, H&E staining). *Source: own*.

**Figure 4:**
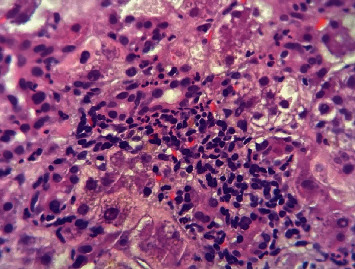
Cholangiolitis. Presence of polymorphonuclear intraepithelial leukocytes in the bile duct (400x, H&E staining). *Source: own*.

Histologically, AFLP is mainly characterized by a diffuse microvesicular steatosis pattern (Figure 1), predominantly in the pericentral area and unusually in the periportal area [9]. Hepatocytes may look normal or pale and swollen (ballooning degeneration) (Figure 2), the cytoplasm is foamy and granular, and the nuclei are centrally located [10]. Other histological findings could be seen, such as bile canalicular plugs (Figure 3) and cholangiolitis (Figure 4), both signs of intrahepatic cholestasis. The accompanying inflammatory infiltrate is usually minimal, and hepatocyte necrosis occurs only in severe cases [10].

The main histopathological differential diagnosis includes pregnancy-induced hypertensive disorders, where the clinical manifestations are similar to AFLP; however, in hypertensive disorders, it is expected to find sinusoidal fibrin deposits or hemorrhage in the periportal areas [11] and in hepatic steatosis due to alcohol, where perivenular and periportal fibrosis, sclerosing hyaline necrosis, venulo-occlusive lesions, and canalicular cholestasis are present [12]; it is important to note that the biopsy findings must be interpreted based on the clinical context of the pregnant patient.

In patients diagnosed with AFLP, morbidity and mortality are secondary to hepatic dysfunction that can affect multiple organs and systems. The time to recovery after delivery depends on the severity of the disease and if there are other complications. The majority of patients will have clinical recovery within 3 to 4 days after delivery, but normalization of laboratory parameters lags frequently [5]. In our case, recovery was reached 9 days after delivery, and the patient required three plasma exchange sessions.

Treatment of AFLP is largely supportive and best carried in a critical care environment with a multidisciplinary team [5]. Artificial liver support therapy (ALST) has been widely used in the management of acute or chronic liver failure caused by various etiologies. This therapy improves liver injury and provides a homeostatic environment for hepatocyte regeneration. Available ALST includes hemoperfusion, plasma exchange (PE), continuous renal replacement therapy, Molecular Adsorbent Recycling System, and Plasma Perfusion (PP). Combining PE and PP in patients with liver disease allows to remove a large number of toxic substances and improve clotting factors and albumin [13]. Some patients will have persistent hepatic failure and acidosis and may be candidates for liver transplantation [14]. Fortunately, our patient recovered normal liver and renal function after PE.

## 4. Conclusion

AFLP is a highly morbid disease that needs a high index of suspicion to be diagnosed. An adequate and timely diagnosis to allow early admission to an ICU to ensure maximum supportive care is essential to reduce maternal mortality. The definitive diagnosis of this disease remains to be made histologically. ALST and PE have been widely recognized as effective therapies in patients complicated by this entity.

## Figures and Tables

**Table 1:** Swansea criteria for AFLP.

Clinical symptoms (i) Nausea and vomiting (ii) Abdominal pain (iii) Encephalopathy (iv) Polydipsia or polyuria
Laboratory findings (i) Bilirubin greater than 0.8 mg/dL (ii) Glucose less than 72 mg/dL (iii) WBC greater than 11 000/microliter (iv) AST or ALT greater than 42 units/L (v) AKI or creatinine greater than 1.7 mg/dL (vi) Ammonia greater than 47 micromoles/L (vii) Coagulopathy or PT greater than 14 s (viii) Urea greater than 340 micromoles/L
Ultrasonographic features (i) Ascites (ii) Echogenic liver
Histologic features (i) Microvesicular steatosis

Source: Nelson et al. [14].

## Abbreviations

AFLP: Acute fatty liver of pregnancy
ALST: Artificial liver support therapy
ICU: Intensive Care Unit
PE: Plasma exchange
PROM: Premature rupture of membranes
PP: Plasma Perfusion.
